# Changes in breeding phenology of eastern Ontario frogs over four decades

**DOI:** 10.1002/ece3.501

**Published:** 2013-02-26

**Authors:** Samantha P Klaus, Stephen C Lougheed

**Affiliations:** Department of Biology, Queen's UniversityKingston, Canada

**Keywords:** Anuran, calling activity, climate change, emergence, precipitation, reproductive timing, temperature

## Abstract

Global climate change has been implicated in phenological shifts for a variety of taxa. Amphibian species in particular are sensitive to changes in their environment due to their biphasic life history and restricted reproductive requirements. Previous research has shown that not all temperate amphibian species respond similarly to the same suite of climatic or environmental cues, nor are individual species necessarily uniform in their responses across their range. We examined both the timing of spring emergence and calling phenology of eight anuran species in southeastern Ontario, Canada, using an approximately 40-year dataset of historical records of amphibian activity. *Rana pipiens* was the only species out of eight considered to emerge significantly earlier, by an estimated 22 days over four decades. Both *R. pipiens* and *Bufo americanus* have advanced initiation of calling over a four-decade span significantly earlier by an estimated 37.2 and 19.2 days, respectively. *Rana sylvatica* showed a trend toward earlier emergence by 19 days, whereas we did not detect changes in emergence phenology for the remaining five species. This significant shift in breeding behavior for two species correlates to significant regional increases in spring temperatures of an estimated 2.7–2.8°C overall over four decades. Our study suggests that local temperature increases have affected the timing of emergence and the onset of calling activity in some Ontario anuran species. Global decline or range shifts ultimately may be related to changes in reproductive behavior and timing mediated by shifting climate.

## Introduction

Climate change will have profound implications for both human welfare and biodiversity in the 21st Century (McCarty 2001; Tol 2002; Parmesan 2006; Hannah 2011). Understanding how climate change impacts ecology, evolutionary trajectories, and distributions of species is a major focus of climate change research (Visser and Both 2005; Lawler et al. 2010; Geyer et al. 2011). Indeed, shifts in phenology of a range of organisms have been attributed to changes in climate (Parmesan 2006; Primack and Miller-Rushing 2011). For example, annual increases in average air temperature have been shown to correlate with earlier initiation of the breeding season for several temperate taxa, including amphibians (Beebee 1995; Blaustein et al. 2001; Brodman 2009) and birds (Brown et al. 1999; Dunn and Winkler 1999), accelerated onset of the growing seasons for many plants (Chmielewski and Rötzer 2001; Stöckli and Vidale 2004), and increasingly synchronous timing of emergence in many insects (Kearney et al. 2010; Westwood and Blair 2010). Changes in phenology can lead to ecological mismatches with species mating, breeding, or developing during suboptimal conditions (Stenseth and Mysterud 2002; Visser et al. 2012) with important implications for local population persistence. Shifts in distribution and changes to the range limits of northern hemisphere species have also been attributed to climate change, with species ranges' expected to increase northward and contract at their southern limits where warming temperatures can lead to harsher living conditions (Parmesan 2006). Long-term ecological consequences of changing climate on temperate ecosystems and biota will potentially be exacerbated by other environmental stressors including increased land conversion for agriculture and urbanization, and the spread of pathogens.

Many amphibians have experienced regional extinctions, distributional changes, and population declines over the past decades and several hypotheses have been advanced to account for these new trends, including climate change (Parmesan 2006; Blaustein et al. 2010). Although it is probable that no single factor underlies all of these events, climate change is certainly a major contributor to amphibian declines (Alford and Richards 1999; Blaustein and Kiesecker 2002; Beebee and Griffiths 2005). Amphibians are sensitive to environmental change due to their semi-permeable skin as well as their usually aquatic reproductive and developmental requirements (Parmesan 2007). Due to the biphasic life history of most species, with both aquatic and terrestrial stages, amphibians are predicted to be more vulnerable to diminution of both quality and extent of terrestrial and aquatic environments, compared with other terrestrial vertebrates (Carey and Alexander 2003). Shifting climate results in the decline of some species due to changes in reproductive behavior and timing (Alford and Richards 1999; Collins and Storfer 2003). For example, mirroring many other taxa, some temperate frog species in recent decades have initiated their breeding seasons earlier, presumably in response to higher mean daily temperatures and the earlier onset of spring (Beebee 1995; Gibbs and Breisch 2001; Oseen and Wassersug 2002; Saenz et al. 2006). Previous studies have often focused on a single wetland, or encompassed time periods of less than 10 years. Such studies may not capture phenological characteristics typical of a region or allow insight into long-term trends in the onset or peak times of breeding (Brodman 2009). Despite the potentially profound implications for population and species persistence, biodiversity levels and patterns, and ecosystem functioning, our knowledge is quite limited about the link between local variation in climate and breeding phenology of many organisms including frogs.

In our study, we test for the relation between multiple abiotic environmental factors and calling activity of eight species of frogs across southeastern Ontario over an approximately 40-year period. For each species, we assess which combination of environmental variables best predicts both spring emergence and calling activity. We hypothesize that early spring breeders will be more sensitive to environmental variation for initiating emergence and calling activity than prolonged breeders as they have a limited time frame to breed within optimal conditions for reproductive success. Our study provides insights into the proximate factors that may control initiation of breeding for southeastern Ontario anurans and allows us to better understand and predict responses to continued climate change.

## Materials and Methods

### Species data collection

Observational records of amphibian activity were obtained through the Natural Heritage Information Centre of Ontario and the Ontario Herpetofaunal Summary (OHS) Atlas (http://nhic.mnr.gov.on.ca). OHS began its active volunteer-based data collation in 1984 and currently collects observational data through an online submission form. They request that the observer report any reptile or amphibian sighting in Ontario and provide information on the number of individuals observed, the time and location of the observation, as well as descriptions of the species and site. The majority of the data from both the center and the atlas are from primary sources and historical records from museum and university collections that have been verified by experts.

Many phenological studies to date have used historical volunteer-based observations, or Citizen Science, to determine correlates of seasonal breeding activity (e.g., Dunn and Winkler 1999; Gibbs and Breisch 2001; Gordo and Sanz 2006). Frogs are good candidates for Citizen Science as each species typically has a unique and easily recognizable advertisement call, meaning that acoustic surveys offer a fairly accurate indicator of male reproductive activity (Crouch and Paton 2002; Steelman and Dorcas 2010). In Ontario, the focus of our study, frog species are few and are distinguishable in their call, coloration, size, and preferred habitat types, facilitating both visual and acoustic species identification.

**Figure 1:**
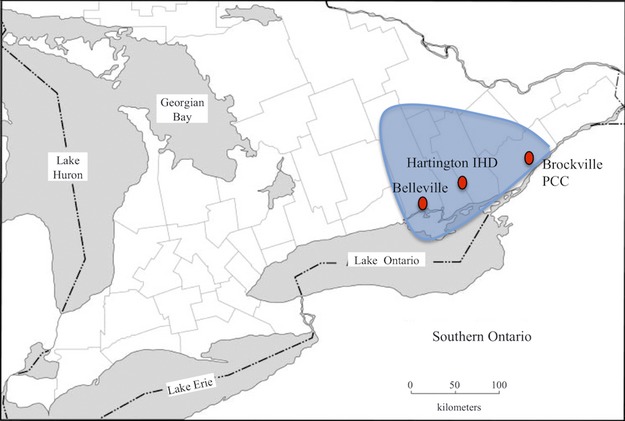
Sampling area (transparent shaded area) from which historical frog observations were obtained for this study. The solid circles within the sampling area indicate the locations of the weather stations used to test long-term trends in temperature and precipitation. IHD: International Hydrological Decade station, PCC: Pollution Control Centre. Map obtained and modified from the Brock University Map Library.

Data were requested for an approximately 200 × 200 km area from southeastern Ontario as this allowed for a reasonably large dataset of observations that included the Queen's University Biological Station (a focus for our phenological studies), while also potentially minimizing confounding spatial effects (de Solla et al. 2006; Fig. 1). Data were available from 1930 to 2010 for nearly all years and for 10 species of anurans, (nine frogs and one toad species). The earliest record in which a species was observed emerging or calling for each year is considered the onset of emergence/calling for that year.

Given that we were interested in breeding phenology, any observations of juveniles or tadpoles were discarded. Observations that did not include a complete date, with day, month and year, or did not include geographic coordinates were also excluded. Years with fewer than five records were eliminated (Dunn and Winkler 1999). Records from the earliest years of data collection were sparse and to minimize data biases, any records that were not within 10 years of another observation were discarded. For our analysis, we only used records of the absolute first day of calling activity or the first day of emergence as indicated by either calling activity or observation for each year and species.

Ultimately, eight anuran species had sufficient data for analysis; American toad (*Bufo americanus*), American bullfrog (*Rana catesbeiana*), chorus frog (*Pseudacris* sp*. –* either *maculatus* or *triseriata*; see below), gray tree frog (*Hyla versicolor*), green frog (*Rana clamitans*), northern leopard frog (*Rana pipiens*), spring peeper (*Pseudacris crucifer*), and wood frog (*Rana sylvatica*). American toads have been placed within the genus *Anaxyrus* and North American members of the genus *Rana* within the genus *Lithobates* (Frost et al. 2006). As *Rana* and *Bufo* are considered scientifically valid by some (Hillis 2007; Pauly et al. 2009) and are the most common names used for these species in previous literature (e.g., Oseen and Wassersug 2002; de Solla et al. 2006; Brodman 2009), we use these naming conventions throughout the article.

*Bufo americanus*, *Pseudacris* sp., *P. crucifer*, and *R. sylvatica* are all early spring breeders that breed in shallow waters. *Hyla versicolor*, *R. clamitans*, and northern *R. pipiens* begin breeding in late spring, whereas *R. catesbeiana* are often the last to begin their breeding season in early summer. *Hyla versicolor* prefer to breed in shallow woodland marshes, whereas *R. clamitans*, *R. pipiens*, and *R. catesbeiana* tend to breed in deeper, permanent bodies of water.

Western chorus frogs (*Pseudacris triseriata*) and boreal chorus frogs (*Pseudacris maculata*) were only relatively recently determined to be genetically distinct species (Platz 1989). The two species have very similar breeding calls and coloration and the extent to which their ranges overlap in its southern Ontario range is currently unknown. Consequently, we cannot be certain of which species of chorus frog is present in the dataset and hereafter refer to chorus frogs as *Pseudacris* sp. None of the aforementioned species are at their northern range limit within the study area.

In this dataset, both *R. pipiens* and *R. catesbeiana* had been documented emerging early in the year when frogs are expected to still be overwintering. Indeed, these species occasionally become active during the core winter months; however, these behaviors are not related to breeding, but instead may be a method of avoiding freezing temperatures and anoxia (Stinner et al. 1994). We thus discard any observations of anurans before February of each year, as well as observations of anurans that were active under the ice surface of water bodies. The resulting pruned dataset comprised approximately 40 years of data between 1970 and 2010 on emergence and the onset of calling activity for all eight focal species.

### Phenology and environment

To examine correlates of breeding phenology, environmental data for each observation were obtained from Canada's National Climate Data and Information Archive, which contains daily historical climate data from both government and university weather stations. We used data obtained from the weather station that was closest to each record's geographic location (Saino et al. 2011). If more than one station was within the same 1-degree latitude and 1-degree longitude (latilong) block, or if the data available from the closest station were incomplete, we used an average of the relevant stations.

As minimum temperatures for the months in which movement occurs may be more insightful for examining behavior of nocturnal species (Todd et al. 2010), environmental variables examined included total monthly precipitation (mm) and minimum monthly air temperature (°C) values for both the most common month of activity for each species, as determined from the data, as well as for the preceding month (Gordo and Sanz 2006). The monthly values were determined from averaged daily values. The maximum air temperature (°C) for the winter months at this latitude (December to February), averaged from daily values, preceding the month of activity each year was also used in analyses. Elevation (*m*) for each record was obtained from an average of all relevant weather stations.

### Annual climatic variation

Three weather stations within the study area with nearly complete and continuous monthly datasets for 1970–2010 were chosen to examine trends in annual climatic variation; Brockville PCC (44°36′00.00″N 75°, 40′00.00″W), Belleville (44°09′02.05″N, 77°23′41.04″W), and Hartington IHD (44°25′41.02″N, 76°41′25.08″W; Westwood and Blair 2010; Fig. 1). All observations were included and were averaged across the three stations to allow for an examination of total monthly precipitation (mm) as well as maximum and minimum monthly air temperatures (°C). Each month was tested independently for long-term patterns.

### Data analyses

Analyses were performed using R (Cran version 2.11-3; R Development Core Team 2011) with an α of 0.05 applied to all tests. Least squares linear regressions of maximum and minimum air temperatures, and total precipitation against year were performed for each month to evaluate long-term climatic variation. A Pearson correlation coefficient was used to determine the relationship between annual first date of emergence and first date of calling activity for each species separately. Linear regression was also used to assess the relationships between first day of emergence or calling activity and each of the environmental parameters, as well as for first day of emergence or calling activity across years. A Cleveland dot plot as well as a visual inspection of autocorrelation functions suggested that days of first activity across years were independent of one another for each species (Crawley 2007; Zuur et al. 2009).

Tests of the assumptions of linear regression were performed using the R package ‘gvlma’ (Pena and Slate 2006) as well as by visual inspection of the residuals. Residuals were tested for normality using a Shapiro-Wilk normality test and variables for which we found departures from normality were log-transformed. For statistical analysis of calling and emergence activity, calendar dates were converted into Julian dates, with Day 1 being January 1st, and accounting for an additional day on leap years (Gibbs and Breisch 2001).

Environmental data were gathered from the weather stations closest to each observation and as such were not comparable among species; Pearson correlation coefficients among the environmental variables were estimated for each species separately. If any coefficient had *r* > 0.5, one of the correlated variables was excluded from the regression analyses (Oseen and Wassersug 2002). Temperature decreases with increasing latitude as well as increasing altitude, and over large distances, this may cause temporal segregation of breeding activity in amphibians across either a latitudinal gradient or between elevations (Parmesan 2007). Latitudinal (43° to 45°) and longitudinal (−75.5° to −77.5°) spans were small in our study, and elevation was relatively consistent across locations (107.0 ± 29.6 m); these three variables were not expected to affect phenology at this spatial scale (de Solla et al. 2006). However, if we found a significant effect of any of these variables when regressed against the first day of activity, they were included in the final analysis.

Multiple linear regressions were performed through backward-stepwise model selection using the Akaike information criterion (AIC), with a significance level of 0.05 required for variables to remain in the model (Salvador and Carrascal 1990). Model fit for each regression was assessed with an α of 0.05 maximum likelihood estimation. Models were tested for low overall residual deviance and normal quantile–quantile distribution (Zuur et al. 2009).

Our ability to detect the onset of breeding or calling may be skewed because most of the data were from volunteer observers that may create variance in sampling effort over years. Using the method described by Gibbs and Breisch (2001), a linear regression of first emergence date between years versus the number of complete records available per year was performed for each species individually to test whether phenological shifts could simply reflect greater sampling intensity in later years. We found no significant effect of number of observations on change in first emergence date between years for any of the test species (*r*^2^ ≤ 0.06, *P* > 0.17), suggesting that our results are not artifacts of sampling intensity bias.

## Results

### Annual climatic variation

Between 1970 and 2010 in southeastern Ontario, average maximum monthly spring temperatures significantly increased; March increased by 0.07°C per annum, or 2.8°C over four decades (*F*_1,39_ = 4.267, *R*^2^ = 0.099, *P* = 0.046), and in April by 0.06°C per annum, or 2.4°C over four decades (*F*_1,39_ = 5.847, *R*^2^ = 0.130, *P* = 0.020). Average maximum monthly fall temperatures also significantly increased; September increased by 0.06°C per annum, or 2.4°C over four decades (*F*_1,39_ = 10.280, *R*^2^ = 0.209, *P* = 0.003), and November increased by 0.05°C per annum, or 2.0°C over four decades (*F*_1,39_ = 5.137, *R*^2^ = 0.116, *P* = 0.029). There was also a significant increase in the average minimum monthly temperatures in April (*F*_1,39_ = 4.267, *R*^2^ = 0.099, *P* = 0.046) and June (*F*_1,39_ = 6.113, *R*^2^ = 0.136, *P* = 0.018) of 0.04°C per annum, or 1.6°C each over a four-decade span (Table S1; Supplementary Material).

There was a significant decrease in average total precipitation for the spring month of March (0.71 mm per annum, 2.84 cm total; *F*_1,39_ = 5.567, *R*^2^ = 0.125, *P* = 0.023) and a significant increase for the summer month of June (0.89 mm per annum, 3.56 cm total; *F*_1,39_ = 4.662, *R*^2^ = 0.107, *P* = 0.037) over 40 years (Table S1; Supplementary Material).

### Abiotic correlates with anuran phenology

*Rana pipiens*, *P. crucifer*, *H. versicolor,* and *R. clamitans* were the species most frequently observed, with an average of 80 observations each year, whereas *R. sylvatica* and *Pseudacris* sp. were the least frequently documented with an average of 40 observations each year. *Rana catesbeiana* had an average of 50 observations and *B. americanus* had an average of 60 observations annually. Observations were relatively evenly distributed across the sampling area.

April was the most common month for both emergence and initiation of calling in five of eight species; *B. americanus*, *R.pipiens*, *P. crucifer*, *Pseudacris* sp*.,* and *R. sylvatica. Rana clamitans* was often first seen in April, but did not begin calling until June. *Rana catesbeiana* and *H. versicolor* were found to emerge in May with *H. versicolor* beginning to call in May and *R*. *catesbeiana* calling in June.

The annual first date of emergence and first date of calling activity were strongly correlated for four species – *B. americanus* (*r* = 0.96), *P. crucifer* (*r* = 0.94), *Pseudacris* sp. (*r* = 0.97), and *R. sylvatica* (*r* = 0.93). There was a weaker correlation between annual first date of emergence and first date of calling activity for the remaining four species – *R. catesbeiana* (*r* = 0.46), *R. clamitans* (*r* = 0.22), *R. pipiens* (*r* = 0.30), and *H. versicolor* (*r* = 0.61)

**Figure 2:**
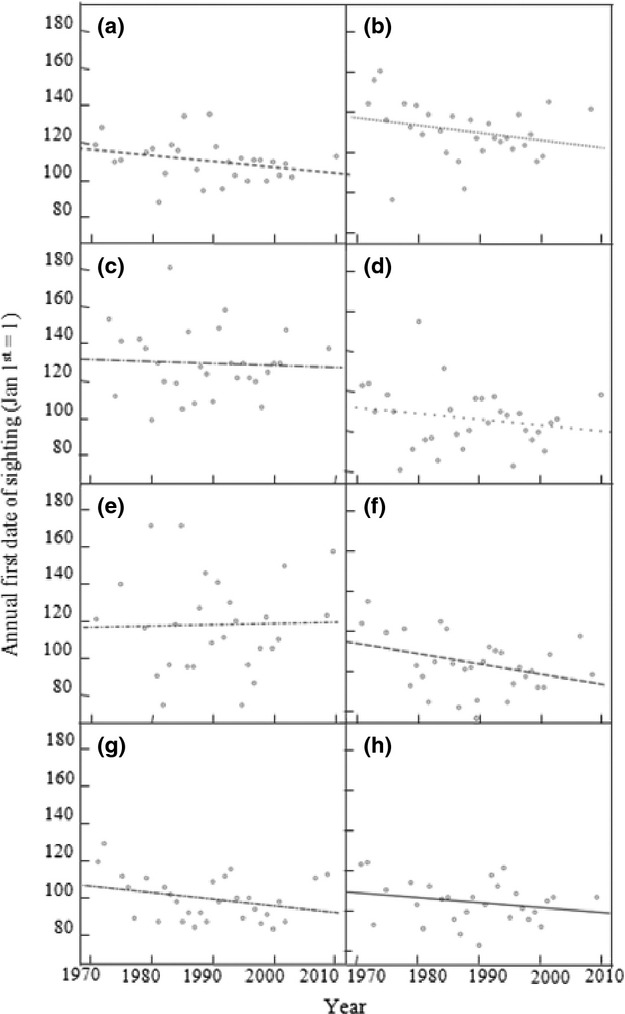
Least squares linear regression trend lines for annual first sighting of southeastern Ontario frog species: (a) *Bufo americanus*, (b) *Hyla versicolor*, (c) *Rana catesbeiana*, (d) *Rana sylvatica*, (e) *Rana clamitans*, (f) *Rana pipiens*, (g) *Pseudacris crucifer*, and (h) *Pseudacris* sp.

*Rana pipiens* was the only species for which the first day of observation shifted significantly earlier, by 0.55 days per year, or 22 days over a four-decade span (*F* = 4.578, *R*^2^ = 0.141, *P* = 0.041, df = 28). The remaining seven species showed no strong trends in dates of first observation (Fig. 2).

**Figure 3:**
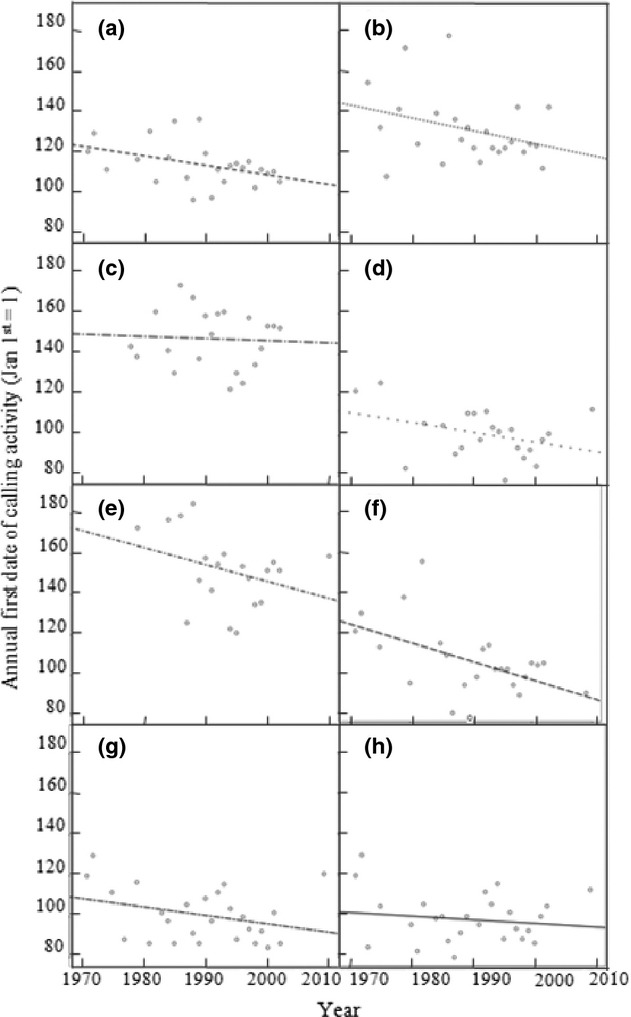
Least squares linear regression trend lines for annual first calling activity of southeastern Ontario frog species: (a) *Bufo americanus*, (b) *Hyla versicolor*, (c) *Rana catesbeiana*, (d) *Rana sylvatica*, (e) *Rana clamitans*, (f) *Rana pipiens*, (g) *Pseudacris crucifer*, and (h) *Pseudacris* sp.

Two anuran species also showed significant shifts in their first day of calling; *B. americanus* were calling earlier by 0.48 days per year (*F*_1*,*22_ = 4.406, *R*^2^ = 0.167, *P* = 0.048), or 19.2 days over four decades, and *R. pipiens* were calling earlier by 0.93 days per year, or 37.2 days over four decades (*F*_1*,*22_ = 8.108, *R*^2^ = 0.269, *P* = 0.009). *Rana sylvatica* appeared to be calling earlier by an average of 0.48 days per year, or 19.2 days over four decades; however, the trend was borderline non-significant (*F*_1*,*20_ = 3.157, *R*^2^ = 0.136, *P* = 0.091). The remaining five species showed no strong trends in dates of first calling activity (Fig. 3).

All six species that were typically first sighted in April had significant whole model relationships with the environmental predictors that we evaluated. First day of sighting for *B. americanus* was negatively correlated with minimum April temperatures, and the first day of sighting for *Pseudacris* sp. was negatively correlated with minimum April temperatures as well as positively correlated with total March precipitation and negatively correlated with maximum winter temperatures. First day of sighting for *R. clamitans* was also positively correlated with total precipitation of March and April as well as negatively correlated with the confounding factor of longitude. First day of sighting for *R. pipiens* was negatively correlated with March minimum temperatures as well as positively correlated with maximum winter temperatures and the confounding factor of elevation. First day of sighting for *P. crucifer* was negatively correlated with minimum temperatures for March and April. First day of sighting for *R. sylvatica* was negatively correlated with minimum temperatures for March, as well as positively correlated with total monthly precipitation for March and April (Table 1).

**Table 1:** Environmental correlates of the annual first sighting of southeastern Ontario frog species. Explanatory variable is referencing the month of activity, or the month previous to the month of activity when stated as such. Significance at an α of 0.05

Species	df	Multiple *R*^2^	Adjusted *r*^2^	F	*P* < 0.05	Month of activity	Explanatory variable	Estimated change	*t*	*P*(>|*t*|, 0.05)
*B. americanus*	1,26	0.256	0.227	8.936	0.006	April	Minimum monthly temperature (°C)	−3.07	−2.99	0.006
*R. catesbeiana*	1,27	0.065	0.030	1.880	0.182	May	Maximum winter temperature (°C)	2.489	1.371	0.182
*Pseudacris* sp.	3,24	0.674	0.634	16.560	4.8E-06	April	Minimum monthly temperature (°C) +	−2.03	−3.81	8.43E-04
							Total previous monthly precipitation (mm) +	0.09	1.69	0.104
							Maximum winter temperature (°C)	−1.65	−2.14	0.043
*H. versicolor*	1,28	0.297	0.245	5.715	0.008	May	Minimum previous monthly temperature (°C) +	−4.49	−3.25	0.003
							Total monthly precipitation (mm)	0.13	1.46	0.156
*R. clamitans*	3,24	0.364	0.285	4.578	0.011	April	Total monthly precipitation (mm) +	0.21	1.924	0.066
							Total previous monthly precipitation (mm) +	0.47	2.308	0.030
							longitude	−22.18	−3.099	0.005
*R. pipiens*	3,26	0.578	0.530	11.880	4.37E-05	April	Minimum previous monthly temperature (°C) +	−4.46	−5.49	9.28E-06
							Maximum winter temperature (°C) +	2.04	1.99	0.058
							elevation (m)	0.21	2.28	0.031
*P. crucifer*	2,27	0.464	0.424	11.68	2.21E-04	April	Minimum monthly temperature (°C) +	−1.82	−1.86	0.073
							Minimum previous monthly temperature (°C)	−1.99	−2.71	0.012
*R. sylvatica*	3,27	0.618	0.575	14.530	7.92E-06	April	Minimum previous monthly temperature (°C) +	−3.67	−5.18	1.90E-05
							Total monthly precipitation (mm) +	0.19	3.46	0.002
							Total previous monthly precipitation (mm)	0.25	3.09	0.005

First day of sighting for *H*. *versicolor* exhibited a significant whole model relationship with minimum April temperatures and total May precipitation. *Rana catesbeiana* showed a negative relation with maximum winter temperatures; however, the trend was not significant (Table 1).

We found significant whole model relationships for all eight species of frogs with their first date of calling activity correlated with at least one of the examined environmental predictors. First date of calling for *B. americanus* and *R. pipiens,* as well as *R. catesbeiana* and *R. clamitans,* was negatively correlated with minimum temperatures for their months of activity, April and June, respectively. First date of calling for *Pseudacris* sp. was negatively correlated with minimum March and April temperatures. First date of calling for *P. crucifer* and *R. sylvatica* was negatively correlated with minimum March temperatures. *Pseudacris crucifer* was also positively correlated with latitude. First date of calling for *H. versicolor* was negatively correlated with maximum winter temperatures as well as the confounding factors of latitude and elevation (Table 2).

**Table 2:** Environmental correlates of the annual first day of calling activity of southeastern Ontario frog species. Explanatory variable is referencing the month of activity, or the month previous to the month of activity when stated as such. Significance at an α of 0.05

Species	df	Multiple R^2^	Adjusted R^2^	F	*P* < 0.05	Month of activity	Explanatory variable	Estimated change	*t*	*P*(>|*t*|, 0.05)
*B. americanus*	1,22	0.310	0.279	9.904	0.005	April	Minimum monthly temperature (°C)	−3.26	−3.15	0.005
*R. catesbeiana*	1,19	0.226	0.185	5.542	0.030	June	Minimum monthly temperature (°C)	−4.48	−2.35	0.030
*Pseudacris* sp.	2,24	0.619	0.587	19.47	9.43E-06	April	Minimum monthly temperature (°C) +	−2.61	−2.61	0.015
							Minimum previous monthly temperature (°C)	−2.26	−3.27	0.003
*H. versicolor*	3,21	0.490	0.417	6.717	0.002	May	Maximum winter temperature (°C) +	−2.62	−1.76	0.093
							Latitude +	−34.19	−3.98	6.81E-04
							Elevation (m)	−0.19	−2.19	0.040
*R. clamitans*	1,18	0.590	0.567	25.890	7.68E-05	June	Minimum monthly temperature (°C)	−10.59	−5.09	7.68E-05
*R. pipiens*	1,22	0.322	0.291	10.430	0.004	April	Minimum monthly temperature (°C)	−5.04	−3.23	0.004
*P. crucifer*	2,23	0.374	0.320	6.868	0.005	April	Minimum previous monthly temperature (°C) +	−2.20	−2.77	0.011
							Longitude	5.04	1.65	0.112
*R. sylvatica*	1,20	0.200	0.160	5.001	0.037	April	Minimum previous monthly temperature (°C)	−2.40	−2.24	0.037

## Discussion

A literature review of 12 studies examining temporal trends in breeding activities of temperate anurans suggests that many have a significant negative correlation between the onset of breeding activities and the average temperature for the month, or the preceding months, in which breeding is initiated (Terhivuo 1988; Beebee 1995; Gibbs and Breisch 2001; Tryjanowski et al. 2003; Ledneva et al. 2004; Kusano and Inoue 2008; Todd et al. 2010). Precipitation is often suggested as an important factor for initiating the onset of breeding in temperate anurans (Beebee 1995; Tryjanowski et al. 2003; Todd et al. 2010); however, rainfall has only been found to be a significant environmental correlate for the timing of breeding in two temperate frog species, the eastern narrowmouth toad (*Gastrophryne carolinensis*) and the ornate chorus frog (*Pseudacris ornata*) (Todd et al. 2010).

Although regional trends for increased monthly, seasonal, or annual air temperatures have been found in all but three of the studies that we reviewed, only 17 of the 32 frog populations examined were shown to breed or appear earlier. Of the three studies that examined environmental correlates with timing of breeding but did not find increased temperatures, two did not examine the significance of long-term climatic trends (Reading 1998; Blaustein et al. 2001) and only one found a pattern of no annual change in air temperature for the month prior to reproduction (Hartel 2008). For black-spotted pond frogs (*Pelophylax nigromaculata* and *Pelophylax porosa*) in Korea (Primack et al. 2009), as well as Fowler's toads (*Bufo fowleri*) in eastern Canada (Blaustein et al. 2001) and some anuran species in England (simply indicated as calling frogs in the naturalist's notes – see Sparks and Carey 1995), the annual first appearance is occurring later than it has in previous years. Of the studies that we found, four frog populations have not altered their onset of calling (Blaustein et al. 2001; Gibbs and Breisch 2001; Hartel 2008), three have not altered their onset of spawning (Beebee 1995; Blaustein et al. 2001), and six have not altered their arrival dates (Reading 1998; Todd et al. 2010).

Significant regional differences in phenology have been reported for some taxa. *Bufo americanus* shows earlier initiation of breeding in Massachusetts (Ledneva et al. 2004) and in our study, but has not changed its breeding phenology in New York (Gibbs and Breisch 2001). *Pseudacris crucifer* was found to breed earlier in both Massachusetts (Ledneva et al. 2004) and New York (Gibbs and Breisch 2001), but not in South Carolina (Todd et al. 2010) and Michigan (Blaustein et al. 2001). The common frog (*Rana temporaria*) has shifted the onset of breeding earlier in Poland (Tryjanowski et al. 2003) and Finland (Terhivuo 1988), but not in Britain (Beebee 1995). These studies suggest not only that there is variation among temperate frog species in their response to recent climate change but also that variation can occur among populations of the same species.

### Annual climatic variation

Similar to climate trends identified by Gibbs and Breisch (2001) in New York and by Beebee (1995) in Britain, our data suggest that southeastern Ontario has increased in its spring minimum and maximum monthly temperatures and also may be increasing in its winter maximum monthly temperatures. Indeed, many studies that have examined temporal trends in frog breeding activity and climate have shown long-term trends toward warmer springs, warmer winters, or both (e.g., Gibbs and Breisch 2001; Ledneva et al. 2004; Primack et al. 2009; Todd et al. 2010). In our study, the spring month of March has also become drier with decreased total precipitation, whereas the summer month of June has increased its total precipitation over the past 40 years.

### Abiotic correlates with anuran phenology

Two spring-breeding anurans in this study have significantly altered their spring activities tracking southeastern Ontario's warming climate. *Rana pipiens* is both emerging and calling earlier now than four decades ago. Although there was a strong correlation between emergence and first date of calling in *B. americanus,* this species has only advanced its date of first calling (Table 2). We do not know why only some Ontario species exhibit altered breeding phenology. Similar to Gibbs and Breisch (2001), we find no consistency in the relation between earlier onset of activities and taxonomic affiliation, seasonal breeding periods, or preferred breeding habitat, factors that might have explained this variation in response among species. Given that our study encompasses a maximum of four decades and that the average lifespan of some of these species (*R. pipiens* ∼ 4 years and *B. americanus* ∼ 5 years), it is possible that this variation in breeding phenology reflects environment-induced plasticity rather than microevolutionary adaptation (Beebee 1995; Gienapp et al. 2007), although we cannot exclude either possibility without further investigation.

Although we attempted to mitigate biases due to geographic distribution, some focal species might have been affected by factors related to latitudinal and longitudinal variation even at this fine geographic scale. *Rana pipiens* emerges later at higher elevations, whereas *H. versicolor* calls earlier at higher latitudes and higher elevations. *Rana clamitans* emerged earlier at more easterly longitudes and *P. crucifer* was found to call earlier at more western locales.

More than half of the species examined have not altered their breeding activity in response to warmer spring temperatures. For some species, particularly those for which summer and winter temperatures may be more important than those in spring, environmental variables important for initiating breeding behavior may not have varied sufficiently in Ontario over the past 40 years to influence the onset of breeding activity. There are environmental variables that are important for initiating breeding activity for some species (e.g., vegetation type, photoperiod) that we did not include in our study, although we did include predictors that are considered relevant to anuran physiology based on previous literature (Table 2).

We make the not unreasonable assumption that anurans that emerge and begin calling earlier are also breeding earlier; amphibians that do not shift their breeding season as spring temperature increases may suffer increased larval mortality from desiccation or inadequate temperature if they begin spawning late in the hydroperiod of ephemeral water bodies (Blaustein et al. 2010). They may also suffer an indirect effect of climate change by experiencing increased larval competition from altered breeding periods of other amphibian species in the local biotic community (Todd et al. 2010). Conversely, species for which the onset of their breeding season remains relatively constant may have greater reproductive success by not reproducing earlier when there is greater environmental variance. In particular, species that breed in non-ephemeral pools may not experience a reproductive advantage if the increase in larval mortality from early-season variation in weather outweighs the benefits of reduced competition from breeding earlier (Loman 2009). For ephemeral breeding anurans, such benefits may be short-lived, however, if drier spring months result in hydroperiods that are too short for larval development and metamorphosis (Brodman 2009; Blaustein et al. 2010).

Our study demonstrates that some species of frogs in Ontario are indeed shifting their dates of emergence and first calling earlier as spring temperatures increased over the last four decades, which may ultimately affect their reproductive success and local persistence. While increasing mean annual temperature is common across temperate localities, not all frog species, nor even conspecific populations, are responding uniformly to such shifts. This cautions against generalizations about the outcome of changing climate on phenology, range limits, or population persistence across anuran taxa. As we only compared dates of first observation and first calling, it would be valuable to expand the scope of study to assess what environmental factors underpin intensity and duration of calling activity and concomitantly breeding for each species. To better predict how temperate frogs will respond to continued climate change, future studies should investigate whether phenological shifts to changing local environments are attributable to plasticity or to selection for earlier breeding, and consider how responses vary within species at smaller regional scales. Evaluating what other environmental factors correlate with the onset, duration and intensity of breeding activity, and at what geographic scale abiotic factors relate to breeding phenology of frogs will help us quantitate likely outcomes of continued climate change.

## Supporting Information

**Table S1.** Long-term trends in monthly temperature and precipitation for southeastern Ontario from 1970 to 2010. Overall estimated change is given for a 40-year span. Transformed variables have non-transformed estimates being reported. Bold text denotes significance at an α of 0.05.
