# Supplementary material for: Changes in breeding phenology of eastern Ontario frogs over four decades
Source: Ecol Evol. 2013 Feb 26;3(4):835–45. doi: 10.1002/ece3.501 (PMC3631398; doi:10.1002/ece3.501)
Supplement: Supplementary file 1 [file ece30003-0835-SD1.docx]

Table S1. Long-term trends in monthly temperature and precipitation for southeastern Ontario from 1970 - 2010. Overall estimated change is given for a 40 year span (df=1,39). Transformed variables have non-transformed estimates being reported. Bold text denotes significance at an ∝ of 0.05.

| Month | Response variable | Estimated change in response per year | Overall estimated change | *F* | multiple *R*^2^ | *P*<0.05 |
| --- | --- | --- | --- | --- | --- | --- |
| January | Average maximum temperature (°C) | 0.06 | 2.4 | 2.969 | 0.071 | 0.093 |
|  | Average minimum temperature (°C) | 0.05 | 2 | 3.200 | 0.076 | 0.081 |
|  | Total precipitation (mm) | 0.33 | 13.2 | 2.484 | 0.060 | 0.123 |
| February | Average maximum temperature (°C) | 0.06 | 2.4 | 3.553 | 0.083 | 0.070 |
|  | Average minimum temperature (°C) | 0.06 | 2.4 | 2.602 | 0.062 | 0.114 |
|  | Total precipitation (mm) | -0.49 | -19.6 | 1.861 | 0.046 | 0.180 |
| March | Average maximum temperature (°C) | 0.07 | 2.8 | 4.267 | 0.099 | **0.046** |
|  | Average minimum temperature (°C) | 0.03 | 1.2 | 0.939 | 0.024 | 0.338 |
|  | Total precipitation (mm) | -0.71 | -28.4 | 5.567 | 0.125 | **0.023** |
| April | Average maximum temperature (°C) | 0.06 | 2.4 | 5.847 | 0.130 | **0.020** |
|  | Average minimum temperature (°C) | 0.04 | 1.6 | 4.267 | 0.099 | **0.046** |
|  | Total precipitation (mm) | 0.31 | 12.4 | 0.545 | 0.014 | 0.465 |
| May | Average maximum temperature (°C) | 0.03 | 1.2 | 1.136 | 0.028 | 0.293 |
|  | Average minimum temperature (°C) | <0.01 | 0.24 | 0.115 | 0.003 | 0.736 |
|  | Total precipitation (mm) | 0.20 | 8 | 0.314 | 0.007 | 0.578 |
| June | Average maximum temperature (°C) | 0.04 | 1.6 | 3.415 | 0.080 | 0.072 |
|  | Average minimum temperature (°C) | 0.04 | 1.6 | 6.113 | 0.136 | **0.018** |
|  | Total precipitation (mm) | 0.89 | 35.6 | 4.662 | 0.107 | **0.037** |
| July | Average maximum temperature (°C) | <0.01 | 0.16 | 0.060 | 0.002 | 0.809 |
|  | Average minimum temperature (°C) | 0.01 | 0.4 | 1.042 | 0.026 | 0.314 |
|  | Total precipitation (mm) | 0.74 | 29.6 | 2.471 | 0.060 | 0.124 |
| August | Average maximum temperature (°C) | 0.03 | 1.2 | 2.514 | 0.061 | 0.121 |
|  | Average minimum temperature (°C) | 0.02 | 0.8 | 1.585 | 0.040 | 0.215 |
|  | Total precipitation (mm) | 0.02 | 0.8 | 0.002 | <0.001 | 0.962 |
| September | Average maximum temperature (°C) | 0.06 | 2.4 | 10.280 | 0.209 | **0.003** |
|  | Average minimum temperature (°C) | 0.02 | 0.8 | 1.987 | 0.048 | 0.167 |
|  | Total precipitation (mm) | 0.45 | 18 | 0.927 | 0.023 | 0.342 |
| October | Average maximum temperature (°C) | 0.02 | 0.8 | 0.659 | 0.017 | 0.422 |
|  | Average minimum temperature (°C) | 0.02 | 0.8 | 0.556 | 0.014 | 0.460 |
|  | Total precipitation (mm) | 0.59 | 23.6 | 1.955 | 0.048 | 0.170 |
| November | Average maximum temperature (°C) | 0.05 | 2 | 5.137 | 0.116 | **0.029** |
|  | Average minimum temperature (°C) | 0.01 | 0.4 | 0.515 | 0.013 | 0.477 |
|  | Total precipitation (mm) | 0.39 | 15.6 | 1.177 | 0.029 | 0.285 |
| December | Average maximum temperature (°C) | 0.05 | 2 | 1.826 | 0.045 | 0.184 |
|  | Average minimum temperature (°C) | 0.08 | 3.2 | 3.454 | 0.081 | 0.071 |
|  | Total precipitation (mm) | -0.53 | -21.2 | 1.541 | 0038 | 0.222 |
